# Notes on Genito-Urinary Therapeutics

**Published:** 1883-02

**Authors:** 


### NOTES ON GENITO-URINARY THERAPEUTICS.

(*Continued from page 31.*)

AMM. MUR. VESICAL.—Pinching and stitching in bladder as far as urethra, when lying.

Frequent urging with frequent urination.

Constant tenesmus, worse at 4 A. M.

Profuse and frequent micturition at night (chiefly Ant. c., Bry., Caus., *Natr. m.*, Nat. sul., Phos., Physo., Phyt., Spig., SULPH., Thuja.)

Increased amount of urine, though drinks less. (Compare with Aurum, Ambra, Merc.)

Urging, yet only few drops pass until next stool, when it flows freely. (See Alum., Mur. ac.)

Must rise at night to urinate, and passes an unusual amount day before menses.

Copious emission of urine, which has a stale smell, yet remains clear.

Deep yellow urine with light, cloudy sediment.

Sediment like clay. (Berb., Kali c., Ign., Mang., Sars., *Sep.*, *Sulph.*, Sul. ac., ZINC.)

SEXUAL.—Stitches and beating in left spermatic cord.

ANT. CRUD. VESICAL.—Tenesmus of bladder, which arouses him from sleep at night.

At night he passes small quantities of urine in an intermitting stream with painful erections.

Frequent and increased urination at night, with much mucus, intense burning in urethra and backache during the emission. (Backache relieved by passing urine: Lyc., Rhus.)

Frequent and profuse urine, with loose stools. (Acon., Agar., Bell., Puls.)

Involuntary (copious) urination, with cough.

Suppression of urine.

PAINS.—Burning when urinating, the urine being mixed with blood, the urethra feels sore to touch; knotty.

Backache, with burning in urethra during the emission of urine.

SEXUAL.—Excited sexual desire, with uneasiness of the whole body, which prevents him from sitting long.

Nightly pollutions, with or without voluptuous dreams.

Itching of penis; of tip of glans.

Biting itching, as from salt, on left side of scrotum.

